# Immunotherapy around the Clock: Impact of Infusion Timing on Stage IV Melanoma Outcomes

**DOI:** 10.3390/cells12162068

**Published:** 2023-08-15

**Authors:** Lisa Gonçalves, Duarte Gonçalves, Teresa Esteban-Casanelles, Tiago Barroso, Inês Soares de Pinho, Raquel Lopes-Brás, Miguel Esperança-Martins, Vanessa Patel, Sofia Torres, Rita Teixeira de Sousa, André Mansinho, Luís Costa

**Affiliations:** 1 Department of Oncology, Hospital de Santa Maria, Centro Hospitalar Universitário Lisboa Norte, 1649-035 Lisboa, Portugalluiscosta.oncology@gmail.com (L.C.); 2 Department of Economics, University College London, London WC1H 0AX, UK; 3 Department of Political Economy, King’s College London, London WC2B 4BG, UK; teresa.estebancasanelles@kcl.ac.uk; 4 Instituto de Medicina Molecular-João Lobo Antunes, Faculdade de Medicina de Lisboa, 1649-028 Lisboa, Portugal; 5 START Lisbon, Hospital de Santa Maria, Centro Hospitalar Universitário Lisboa Norte, 1649-035 Lisboa, Portugal

**Keywords:** cancer, chronobiology, circadian, immunotherapy, melanoma

## Abstract

Although the impact of circadian timing on immunotherapy has yet to be integrated into clinical practice, chronoimmunotherapy is an emerging and promising field as circadian oscillations are observed in immune cell numbers as well as the expression of immunotherapy targets, e.g., programmed cell death protein-1 and its ligand programmed death ligand 1. Concurrent retrospective studies suggest that morning infusions may lead to higher effectiveness of immune checkpoint inhibitors in melanoma, non-small cell lung cancer, and kidney cancer. This paper discusses the results of a retrospective study (2016–2022) exploring the impact of infusion timing on the outcomes of all 73 patients with stage IV melanoma receiving immunotherapy at a particular medical center. While the median overall survival (OS) was 24.2 months (95% confidence interval [CI] 9.04–39.8), for a median follow-up of 15.3 months, our results show that having more than 75% of infusions in the afternoon results in shorter median OS (14.9 vs. 38.1 months; hazard ratio 0.45 [CI 0.23–0.86]; *p* < 0.01) with more expressive impacts on particular subgroups: women, older patients, and patients with a lower tumor burden at the outset of immunotherapy. Our findings highlight the potential benefits of follow-up validation in prospective and translational randomized studies.

## 1. Introduction

Malignant melanoma affects all age groups and is the leading cause of death from cutaneous malignancies, accounting for more than 20,000 deaths annually in Europe [1]. The disease has markedly distinct prognoses according to the disease stage. In stages I–II, the 5-year overall survival (OS) ranges between 90 and 100%; stage III has a 5-year OS of approximately 75%; and stage IV (metastatic melanoma) has a 5-year OS of 9–35% according to the disease burden, lactate dehydrogenase (LDH) level, presence of central nervous system (CNS) metastases, and metastatic involvement of multiple organs. In addition, other factors have been shown to potentially influence prognosis, namely inflammatory markers such as peripheral blood neutrophil/lymphocyte ratio and platelet count. These factors independently contribute to the systemic inflammatory index that correlates with worse outcomes [2].

Immunotherapy is currently the standard of care in metastatic melanoma treatment [3]. Before 2011, therapeutic choices for metastatic melanoma were limited, and the median OS was 9 months [4]. In 2011, the emergence of immune checkpoint inhibitors (ICIs) revolutionized the treatment landscape of metastatic melanoma, a malignancy documented as highly immunogenic due to high levels of tumor-infiltrating lymphocytes [5]. Immune checkpoints downregulate the immune system, preventing its overactivation, and are used by cancer cells as an escape mechanism from the immune system [6,7]. Despite frequently reported high-grade toxicity [8], ipilimumab—a fully humanized immunoglobulin G1 (IgG1) anti-cytotoxic lymphocyte antigen-4 (CTLA-4)-blocking antibody—was the first ICI approved for the first-line treatment of stage IV melanoma. In 2015, other ICIs emerged, targeting the negative regulation of T cell activation through the blockade of the programmed cell death protein-1 (PD-1)/programmed death ligand 1 (PD-L1) pathway. Inhibition of this pathway activates the host’s immune response. This led to a significant survival benefit in metastatic melanoma, extending the 12-month OS associated with the PD-1 inhibitors pembrolizumab and nivolumab to over 70% [9,10,11,12]. Recently, combined ICI with an anti-CTLA-4/anti-PD-1 induction phase followed by an anti-PD-1 maintenance phase (ipilimumab/nivolumab) was approved in metastatic melanoma, following results of 72.1 months of median OS versus 36.9 months with anti-PD-1 nivolumab, and 19.9 months with anti-CTL-4 ipilimumab monotherapies [13]. Notwithstanding the significant progress, the prognosis of these patients remains dismal, with a 5-year OS just over 50% with the anti-CTLA-4/anti-PD-1 combination [13,14], with a considerable number of patients presenting as non-responders.

The interplay between cancer cells and the tumor microenvironment affects cancer cell survival, local invasion, and metastatic dissemination [15]. The tumor microenvironment comprises several non-cancer cells, growth factors, cytokines, and the extracellular matrix. Tumor microenvironment cells predominantly consist of fibroblasts, epithelial cells, and immune cells such as lymphocytes, natural killer (NK) cells, tumor-associated macrophages (TAMs), myeloid-derived suppressor cells, dendritic cells (DCs), and tumor-associated neutrophils. Immune cells from the tumor microenvironment infiltrate the tumor and can elicit both tumorigenic and anti-tumorigenic effects, playing a decisive role in tumor growth and therapy response [15]. The tumor microenvironment has a high heterogeneity of cells, which vary according to tumor type, making each tumor microenvironment unique [15,16].

The immune landscape varies throughout the day, suggesting an impact on tumor proliferation. Immune cells, NKs, DCs, monocytes, and T and B lymphocytes exhibit circadian oscillations in the peripheral blood [17,18,19,20]. For instance, the number of circulating CD4+ and CD8+ T cells doubles between early morning and early night [17]. Wang et al. [21] recently showed more aggressive tumor behavior in mice engrafted with melanoma cells in the evening versus in the late afternoon, as well as variation in the anti-tumorigenic activity of CD8+ T cells according to the time of day. The authors also showed that mice with melanoma cells inoculated during the day had a higher DC count and better anti-tumor response, with greater tumor volume suppression, than those that were vaccinated during the night. CD8+ T cell clones from melanoma patients exhibited different T cell proliferation abilities according to the time of day [21]. Additionally, other studies demonstrated that peripheral blood immune cells and their migration to organs exhibit an oscillating off-phase pattern. These cells predominantly exit hematopoietic organs and enter the peripheral blood during the onset of the behavioral rest phase. Conversely, they migrate predominantly to peripheral organs during the onset of the behavioral active phase [22].

The PD-1/PD-L1 expression levels of ICIs’ targets fluctuate throughout the day, which may result in different ICI efficacy according to the timing of administration. The ICIs nivolumab and pembrolizumab, used in the treatment of metastatic melanoma, act by blocking the interaction of PD-1 with PD-L1/2, thereby inhibiting the PD-1 pathway and promoting immune system activation [7]. Besides being present in T cells, the PD-1 receptor is also expressed on TAMs and DCs from the tumor microenvironment, and its expression levels increase as cancer progresses [23]. Furthermore, the presence of PD-1 reduces phagocytosis and thus limits the ability of macrophages to eliminate cancer cells, which enhances the immune escape of tumor cells [24]. In a melanoma-bearing mouse model, circadian oscillations were reported in PD-1, *Pdcd1*, and in the PD-L1-encoding *Cd274* gene [25]. Indeed, Tsuruta et al. [25] showed that circadian PD-1 expression on TAMs impacts the anti-tumor effect of the PD-1/PD-L1 inhibitor BMS-1 in melanoma-bearing mice, with tumor growth significantly suppressed by the administration of BMS-1 during the night. Tumor cells usually express PD-L1 on their surfaces; however, the expression of PD-1 in tumor cells has also been reported. The PD-1 mechanism in tumor cells is not yet clear, and it seems to differ between tumor types [23].

A few concurrent retrospective studies suggest an impact of circadian timing on treatment effectiveness in different metastatic settings. In metastatic melanoma, the MEMOIR retrospective study showed that administering more than 20% of ICI (ipilimumab, nivolumab, and pembrolizumab) infusions later than 4:30 p.m. was associated with worse outcomes [26]. In non-small cell lung cancer (NSCLC), Kabaroué et al. [27] report a retrospective study in metastatic NSCLC that indicates a major OS difference according to the immunotherapy infusion timing, with patients receiving morning infusions showing a four-fold OS increase compared to those receiving afternoon infusions. In metastatic renal cell carcinoma, two concurrent retrospective studies [28,29] showed that morning immunotherapy infusions were again associated with better objective response rates, time to treatment failure, and OS. A small-sample, pan-cancer retrospective study did not find significant OS differences between performing ICI infusions in the morning or late afternoon [30]. However, it is unclear if the allocation into morning and afternoon groups across patient characteristics and tumor types correlated with the effectiveness of the treatment, which could explain the lack of a significant effect. Additionally, when controlling for the number of infusions, Cortellini et al. [31] failed to find a significant association between evening infusions and aggravated OS. Because the number of infusions is also a result of treatment success itself, controlling for it entails an indirect selection on outcomes, which may explain the absence of statistically significant differences. These data stress the need for further studies.

This paper reports on a retrospective study of patients with metastatic melanoma. We contribute to the growing body of evidence that suggests a significant beneficial impact of performing immunotherapy in the morning on patient outcomes. Further, we find suggestive evidence of more expressive positive impacts of morning infusions on particular subgroups: women, older patients, and patients with a lower tumor burden at the start of immunotherapy. Prospective studies and translational approaches are needed to further our understanding of the potential gains and mechanisms underlying these results.

## 2. Materials and Methods

### 2.1. Study Design

We perform a retrospective cohort study of patients with stage IV melanoma receiving immunotherapy with either nivolumab, pembrolizumab, or ipilimumab plus nivolumab, in first or later lines of treatment, at the medical center ‘Centro Hospitalar Universitário Lisboa Norte’ in Portugal between July 2016 and March 2022. Inclusion in the sample was restricted to patients with an Eastern Cooperative Oncology Group (ECOG) performance status (PS) of 0–1 at the start of immunotherapy.

### 2.2. Participants

**Figure 1:**
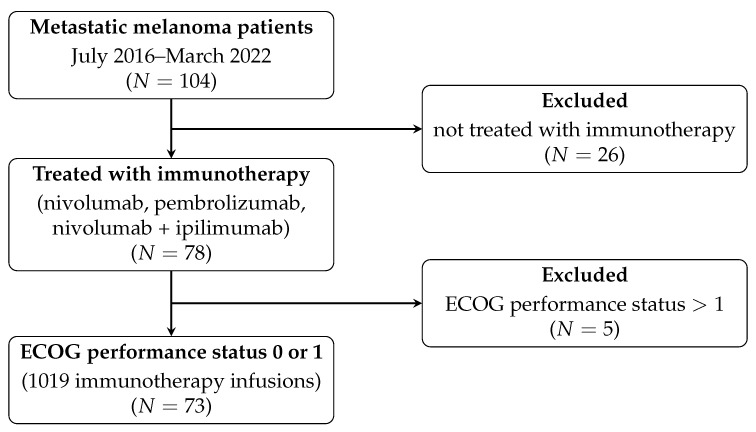
Patient Inclusion Criteria. ECOG: Eastern Cooperative Oncology Group.

Our dataset comprises 104 patients with metastatic melanoma, of which 78 received immunotherapy with either nivolumab, pembrolizumab, or nivolumab plus ipilimumab. Out of these, only 73 patients had an ECOG PS of 0–1 at the start of immunotherapy. This patient cohort completed a total of 1019 infusions between July 2016 and March 2022 (Figure 1). All data were pseudoanonymized.

### 2.3. Outcomes

Data on demographic and clinical–pathological characteristics, infusion reactions, immune-related adverse events (irAEs), and timing of administration of each immunotherapy cycle were retrieved from the patients’ medical records. Adverse events were categorized according to the Common Terminology Criteria for Adverse Events (CTCAE-Version 5.0) [32]. Disease progression was determined based on RECIST 1.1 (Response Evaluation Criteria in Solid Tumors) criteria [33] or clinical assessment.

### 2.4. Treatment Groups and Allocation

Infusion times were retrieved from medical records and split into two treatment groups: the AM (morning) treatment group (8 a.m.–2 p.m.) and the PM (afternoon) treatment group (2 p.m.–8 p.m.). The AM group included all patients who received less than 75% of infusions after 2 p.m., and the PM group included all patients who received at least 75% of infusions after 2 p.m. Although no explicit randomization device was used to allocate patients to study groups, we verify that neither patients’ baseline characteristics nor initial disease burden correlate with treatment allocation. On this basis, we argue that the estimates presented in this paper may be interpreted as causal. Note that the potential identification of a causal relationship is independent of the inference-related limitations posed by the relatively small sample used.

### 2.5. Statistical Analysis

Tests of independence of treatment allocation from patient characteristics and initial disease burden relied on $\chi^{2}$ independence tests. Time-to-event outcomes were analyzed using Kaplan–Meier nonparametric estimators, and statistical inference tests were based on Cox regression analysis. All tests were conducted considering a two-sided 5% significance level.

## 3. Results

### 3.1. Patient Characteristics and Disease Burden by Treatment Group

Patient characteristics at the start of immunotherapy are summarized in Table 1. A majority of patients in the study cohort were male (62%, *N* = 45). The median age at diagnosis was 64 years (range 25–89 years), and 70 years (range 29–91 years) at the start of immunotherapy. Most patients (85%; *N* = 62) presented a cutaneous melanoma subtype; the remaining presented rare melanoma subtypes—e.g., mucosal (12%; *N* = 9) and ocular melanoma (3%; *N* = 2).

Regarding the patients’ disease burden (Table 2), 32% (*N* = 23) had lesions in a single metastatic site, 37% (*N* = 27) in two metastatic sites, 23% (*N* = 17) in three metastatic sites, and 8% (*N* = 6) in four or more metastatic sites. CNS metastases were present in 19% (*N* = 14) of cases. Patients’ median LDH level before the start of the immunotherapy was 410 U/L (range 117–4529 U/L), with 42% (*N* = 31) showing a level above the upper limit of normal (ULN), and 15% (*N* = 11) twice above the ULN (set at 500 U/L).

Of the 73 patients in the sample, 66% (*N* = 48) were allocated to the AM treatment group (<75% of infusions in the afternoon) and 34% (*N* = 25) to the PM group (≥75% of infusions in the afternoon). The two treatment groups were mainly composed of male patients who were 70 or older at the start of immunotherapy, and patients predominantly with an ECOG PS of 0, with no statistically significant differences between both regarding clinical–demographic features (Table 1). Also, no significant differences were found between the treatment groups regarding disease burden (Table 2), and number of immunotherapy infusion sessions (median of 15 [range 2–44] vs. 13 [range 2–59] in the AM and PM treatment groups, respectively).

### 3.2. Immunotherapy Toxicities

The toxicities associated with immunotherapy and respective grades (CTCAE v5.0) are summarized in Table 3. The majority of patients in the study experienced at least one instance of irAEs (66%, *N* = 48). The most common irAE was fatigue (*N* = 27, 37%), followed by cutaneous (*N* = 24, 33%), endocrine (*N* = 16, 22%), and renal (*N* = 8, 11%) toxicities. Cutaneous toxicities consisted of rash, pruritus, and vitiligo; endocrine toxicities denotes hypothyroidism, hyperthyroidism, thyroiditis, adrenal insufficiency, and hypophysitis.

Grade 3–4 irAEs were only reported in the AM treatment group. Despite this, no statistically significant differences were found in overall, G1/G2, or G3/G4 irAEs between both treatment groups. Severe irAEs included endocrine toxicity (hypothyroidism, *N* = 2), hepatitis with transaminase elevation (*N* = 2), cutaneous toxicity (rash, *N* = 1), uveitis (*N* = 1), pneumonitis (*N* = 1), and encephalitis (*N* = 1). Three patients suspended immunotherapy due to toxicity.

### 3.3. Progression-Free and Overall Survival

**Figure 2:**
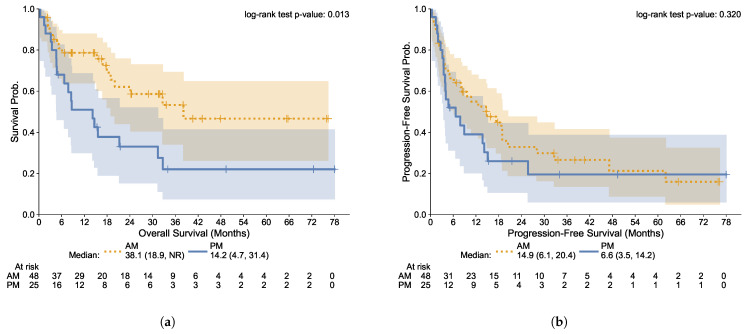
Kaplan–Meier Curves of Overall and Progression-Free Survival by AM/PM Treatments. (**a**) Overall Survival; (**b**) Progression-Free Survival. AM (Morning) group: patients with <75% of infusions after 2 p.m.; PM (Afternoon) group: patients with ≥75% of infusions after 2 p.m. Lines: Kaplan–Meier nonparametric estimates of survival rates; shaded regions: 95% confidence intervals.

The median progression-free survival (PFS) of the study cohort was 10.7 months (95% CI 4.0–17.5) and the median OS was 24.2 months (95% CI 9.0–39.3), for a median follow-up of 15.3 months. There was a trend toward higher PFS in the AM treatment group (median 14.9 months [95% CI 6.1–20.4] vs. 6.6 months [95% CI 3.5–14.2] in the PM group), although not reaching statistical significance (*p* = 0.320). Overall survival (OS) was strikingly higher in the AM group: a median of 38.1 months (95% CI 18.9-not reached) versus 14.2 months (95% CI 4.7–31.4) in the afternoon group, with a hazard ratio (HR) of 0.45 (95% CI 0.23–0.86; *p* < 0.01; Figure 2).

### 3.4. Subgroup Analysis

**Figure 3:**
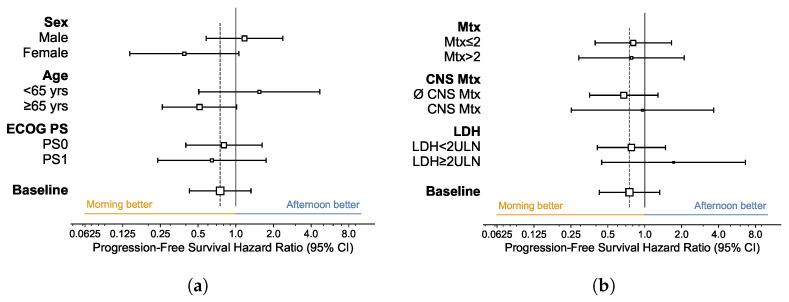
Forest Plot for Hazard Ratio of Progression-Free Survival. (**a**) By Patient Characteristics; (**b**) By Tumor Burden. AM (Morning) group: patients with <75% of infusions after 2 p.m.; PM (Afternoon) group: patients with ≥75% of infusions after 2 p.m. Hazard ratio of AM vs. PM treatment groups estimated using Cox regressions; squares: estimated values (size proportional to sample size); whiskers: 95% confidence intervals. ECOG PS: Eastern Cooperative Oncology Group performance status. Mtx: number of metastatic sites. (∅) CNS Mtx: presence (absence) of metastases in the central nervous system. LDH: lactate dehydrogenase; ULN: upper limit of normal.

**Figure 4:**
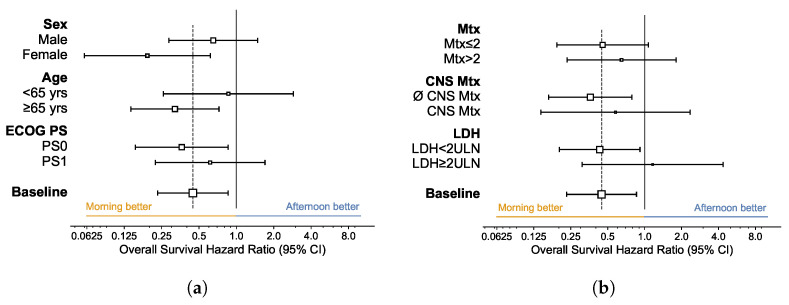
Forest Plot for Hazard Ratio of Overall Survival. (**a**) By Patient Characteristics; (**b**) By Tumor Burden. AM (Morning) group: patients with <75% of infusions after 2 p.m.; PM (Afternoon) group: patients with ≥75% of infusions after 2 p.m. Hazard ratio of AM vs. PM treatment groups estimated using Cox regressions; squares: estimated values (size proportional to sample size); whiskers: 95% confidence intervals. ECOG PS: Eastern Cooperative Oncology Group performance status. Mtx: number of metastatic sites. (∅) CNS Mtx: presence (absence) of metastases in the central nervous system. LDH: lactate dehydrogenase; ULN: upper limit of normal.

We analyzed how the relative effectiveness of allocation to AM vs. PM treatment group varies across patient subgroups. Figure 3 and Figure 4 respectively exhibit hazard ratio (HR) forest plots of PFS and OS across AM and PM treatment groups within different subgroups, as defined by patient characteristics and initial disease burden. In no case was allocation to the AM treatment group detrimental to the effectiveness of immunotherapy.

The data suggest higher relative effectiveness of AM vs. PM treatment allocation on PFS for (i) female patients (median 32.6 vs. 5.7 months; *p*-value 0.056), and (ii) patients aged 65 and older (median 14.6 vs. 5.3 months; *p*-value 0.052). However, the HR is not significantly different from 1 at a two-sided 5% significance level in any of the subgroups (Figure 3).

**Figure 5:**
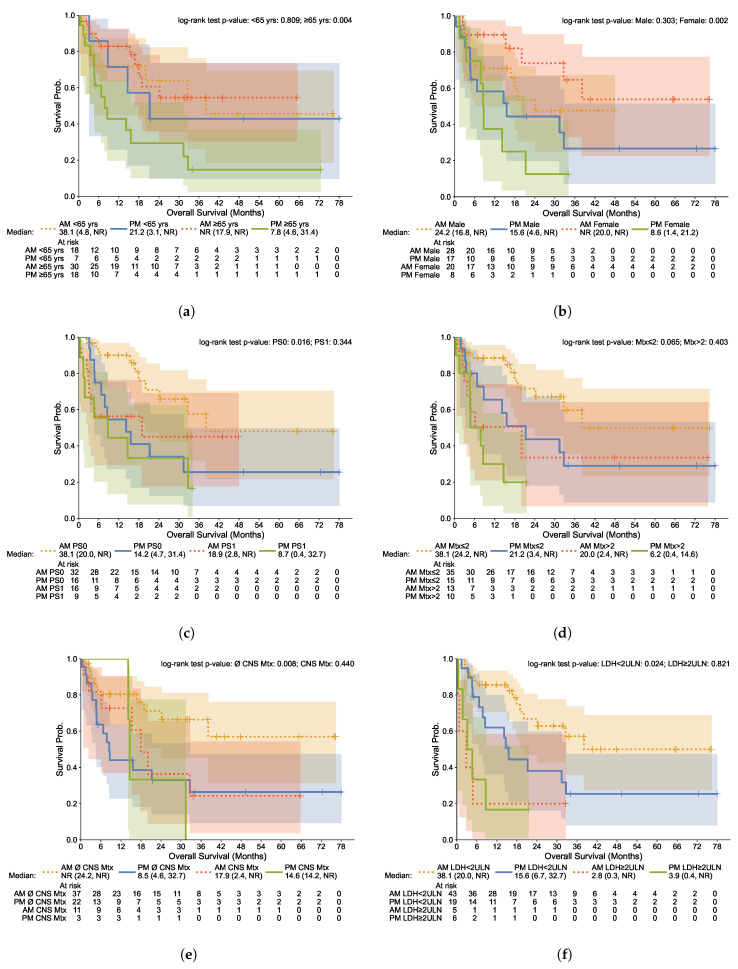
Kaplan–Meier Curves for Overall Survival by AM/PM Treatments: Patient Characteristics and Disease Burden. (**a**) By Age; (**b**) By Sex; (**c**) By ECOG PS; (**d**) By Number of Metastatic Sites; (**e**) By Presence of CNS Metastases; (**f**) LDH. AM (Morning) group: patients with <75% of infusions after 2 p.m.; PM (Afternoon) group: patients with ≥75% of infusions after 2 p.m. Lines: Kaplan–Meier nonparametric estimates of survival rates; shaded regions: 95% confidence intervals. ECOG PS: Eastern Cooperative Oncology Group performance status. Mtx: number of metastatic sites. (∅) CNS Mtx: presence (absence) of metastases in the central nervous system. LDH: lactate dehydrogenase; ULN: upper limit of normal.

Regarding the relative effectiveness of AM treatment allocation on OS, we detect meaningful differences across subgroups. Not only is AM vs. PM treatment allocation conducive to significant effects on HR for (i) female patients (HR 0.19 [95% CI 0.06–0.62]), and (ii) older patients (HR 0.31 [95% CI 0.14–0.72])—and not for male and younger patients—but we also find a significant effect for patients with an initial low tumor burden (fewer metastatic sites [HR 0.45; 95% CI 0.19–1.07]), less CNS involvement [HR 0.36; 95% CI 0.17–0.79], and LDH below 2ULN [HR 0.43; 95% CI 0.20–0.92]), but not for those with worse disease burden at the outset of immunotherapy. For completion, in Figure 5 we report the Kaplan–Meier estimates of OS disaggregated by patient characteristics (age (a), sex (b), and ECOG PS (c)) and initial disease burden (number of metastatic sites (d), presence of metastases in the central nervous system (e), and LDH values (f)).

### 3.5. Robustness Analysis: Varying the Proportion Cutoff of Afternoon Infusions

**Figure 6:**
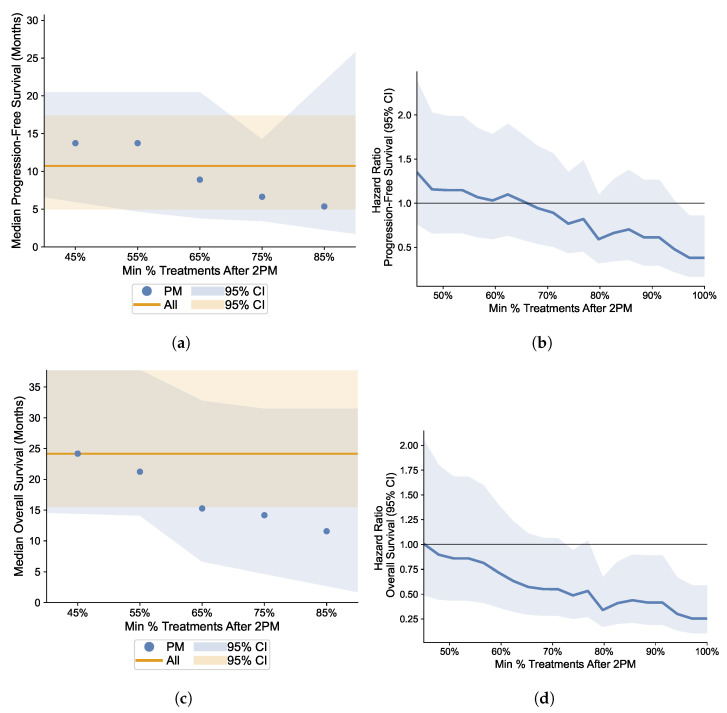
Impact of Fraction of PM Treatments. (**a**) Median Progression-Free Survival; (**b**) Hazard Ratio for Progression-Free Survival; (**c**) Median Overall Survival; (**d**) Hazard Ratio for Overall Survival. AM (Morning) group: patients with <75% of infusions after 2 p.m.; PM (Afternoon) group: patients with ≥75% of infusions after 2 p.m. Lines in panels (**a**,**c**): Kaplan–Meier nonparametric estimates of median PFS (**a**) and median OS (**b**) with varying cutoffs for PM treatment group (*x*-axis); shaded regions: 95% confidence intervals. Lines in panels (**b**,**d**): Cox regression estimates of hazard ratios for PFS (**b**) and OS (**d**) comparing AM vs. PM treatment groups with varying cutoffs for PM treatment group (*x*-axis); shaded regions: 95% confidence intervals.

We explore the effect of varying the cutoff threshold for the proportion of PM infusions underlying the definition of the treatment groups on the relative effectiveness of immunotherapy. The results suggest that the higher the proportion of afternoon immunotherapy infusions used in the definition of the PM treatment group, the less favorable is the outcome relative to the AM group. Panels (a) and (c) of Figure 6 depict the median PFS and OS, respectively, for the entire sample and for varying cutoff proportions of afternoon infusions; the data suggest a negative relation. Panels (b) and (d) of Figure 6 exhibit an analogous exercise, but considering instead the hazard ratio; we find a clear we find a clear downward trend.

## 4. Discussion

### 4.1. Concurrent Research on Chronoimmunotherapy

Central and peripheral circadian clocks have been shown to modulate tumor response and chemotherapy adverse effects [25,34,35,36,37,38,39], and yet they are not routinely used in clinical practice. It was not until recently that the potential benefits of circadian timing of immunotherapy have been the subject of scientific study.

A number of concurrent retrospective studies have sought to estimate the effect of immunotherapy infusion timing on several tumor types. In metastatic melanoma, the MEMOIR retrospective study first documented a potential effect of circadian timing of the administration of immunotherapy (specifically ipilimumab, nivolumab, and pembrolizumab). The authors find that patients who received more than 20% of infusions after 4:30 p.m. had worse OS outcomes (HR of 2.04) [28]. Karaboué et al. analyzed the impact of nivolumab administration timing in NSCLC using the median clock hour of a patient’s treatments and dichotomizing patients into AM (morning) and PM (afternoon) groups according to the period in which they received the most treatments. The authors found that the AM group had a median PFS and OS around four times larger than that of the PM group [27]. Our findings support these results while also providing evidence that allocation to a treatment group is balanced across observables and is, therefore, unlikely to be driving our results. Finally, in metastatic renal cell carcinoma, a correlation has been found between daytime administration of immunotherapy and OS favoring morning infusions [28,29].

Other studies do not find any statistically significant effects of the timing of immunotherapy. A pan-cancer retrospective study [30] in patients with head and neck cancer, triple-negative breast cancer, ovarian cancer, melanoma, and other solid tumors does not find a statistically significant difference in PFS or OS between those who had more than 20% of pembrolizumab infusions after 4:30 p.m. and those who had less. This study also finds no significant differences in treatment outcomes when the cut-off was set to the median time of administration for all pembrolizumab infusions. This could be explained by the fact that INSPIRE is a multi-tumor study, with over 30% being rare solid cancers. Tumor immunogenicity varies greatly between different types of cancer, leading to different responses to immunotherapy. The differences may be difficult to determine in this pan-tumor setting. Cortellini et al. [31] also show positive effects of morning infusions, which are statistically significant at 10%. After controlling for the number of infusions when using propensity score matching, the effect loses its significance. However, because the number of infusions is an outcome variable, using it to match patients in the control and treatment groups is, in fact, selection on outcomes of past treatments. This generates a clear endogeneity issue and could therefore lead to biased estimates.

Overall, the findings of recent studies as well as our own indicate that periodic immunotherapy infusions in the morning may be more effective in metastatic melanoma, NSCLC, and renal cell carcinoma than afternoon infusions. In particular, we find that having less than 75% of infusion sessions in the morning leads to a two-fold increase in the patients’ OS on average. However, we do not find similar effects when using PFS.

Despite our small sample size, we conduct an exploratory heterogeneity analysis to assess whether patients with different characteristics derive the same benefit from immunotherapy morning infusions. Women, older patients, and patients with low tumor burden appear to benefit the most. Moreover, the data suggest a correlation between the progressive increase in the number of treatments performed in the afternoon and the PFS and OS worsening.

No significant differences were found between the AM and PM treatment groups regarding common prognostic factors (age, serum LDH, and ECOG PS). Notwithstanding this, the retrospective nature of this study is a limitation that should be acknowledged, as well as its small sample size, and residual bias in patient allocation to morning and afternoon sessions cannot be excluded.

### 4.2. Potential Underlying Mechanisms

The mechanisms through which the time of immunotherapy administration has an impact on treatment effectiveness remain unclear and should be investigated in follow-up studies. Both anti-CTL4 (ipilimumab) and anti-PD-1 (pembrolizumab and nivolumab) have half-lives of over two weeks [40]. This limits the role of pharmacokinetics in the drugs’ effectiveness when they are administered with different timings, suggesting a role for pharmacodynamics. Furthermore, the effectiveness of the immune response against cancer relies on a plethora of mechanisms, some of which are circadian-dependent [34,36]. For instance, levels of both melatonin—which inhibits one of the main mechanisms of cancer dissemination, the epithelial-to-mesenchymal transition—and cortisol—which can act as both immunoenhancer and immunosuppressive—have a wide range of circadian variation (reviewed in Cortés-Hernández et al. [37]). Women and older people exhibit higher circadian amplitude and higher levels of both plasma melatonin and cortisol compared to men and younger people, respectively [41,42]. These variations can contribute to response patterns inherent to specific demographic groups. Immune cells, such as circulating T and B lymphocytes and dendritic cells, display circadian oscillations in peripheral blood. For instance, lymphocyte migration through lymph nodes is also known to occur in a circadian manner [17,18,19]. Vaccination data showed that immunization in the morning often leads to more competent responses, implying a more targetable immune system in the morning [43,44]. Not only do the levels of immune cells oscillate, but circulating tumor cells (CTCs) also present circadian oscillations [45]: Diamantopoulou et al. [38] recently reported that CTCs in breast cancer preferentially led to metastization during the night.

One study in a mouse model using human malignant melanoma clones showed a circadian-dependent DC and CD8+ T cell anti-tumorigenic effect and proliferation, and immunization during the day led to increased tumor volume suppression [21]. The expression of both PD-1 and PD-L1 varies in a circadian fashion, which further supports the role of circadian rhythm in both tumor and microenvironment biology. As Tsuruta and coauthors reported [25], administering BMS-1—a small-molecule inhibitor of PD-1/PD-L1—at the time of day when PD-1 expression is higher on TAMs increases its anti-tumor activity, suggesting that adjustment of the most appropriate time of day to administer ICIs may take into consideration the circadian expression of PD-1 on TAMs. ICIs are the standard of care in metastatic melanoma, and led to a significant increase in OS, with a median OS of over 5 years [46].

**Figure 7:**
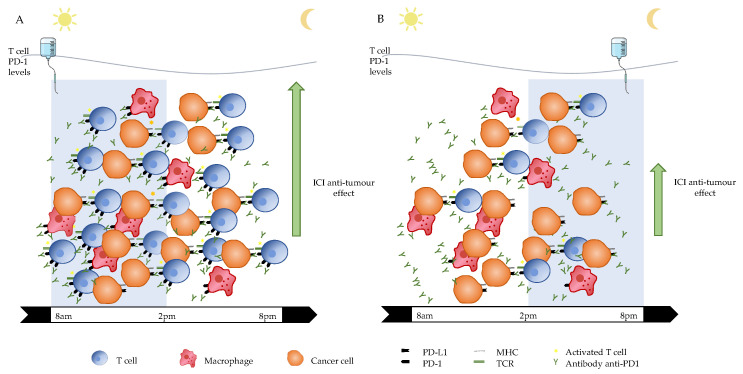
Hypothetical Model for Differences of ICI Anti-Tumor Effect according to the Circadian Timing of Infusion. Panel (**A**): hypothetical immune setting and ICI anti-tumor effect during the morning. Panel (**B**): hypothetical immune setting and ICI anti-tumor effect during the afternoon. ICI: immune checkpoint inhibitor. PD-1: programmed death protein 1. PD-L1: programmed death ligand 1. MHC: major histocompatibility complex. TCR: T cell receptor.

Here we hypothesize a mechanism which implies circadian variations of both T cell number and PD-1 expression levels, with lower T cell numbers and PD-1 expression levels in the afternoon (Figure 7). We suggest that, upon exposure to ICIs, the higher the T cell number and PD-1 expression, both on T cells and TAMs, the more efficient the ICIs, leading to increased activation and expansion of effector T cells and, consequently, to more competent tumor suppression and disease control.

## 5. Conclusions

Data continue to emerge on the multitude of mechanisms through which the circadian timing of immunotherapy infusion may lead to better treatment outcomes. This unlocks the possibility of changing the disease’s course just by changing the timing of treatment administration, which may deliver substantial gains at low cost.

While this paper’s findings provide valuable insights into the potential role of the circadian timing of ICI treatments for metastatic melanoma, prospective randomized studies with a translational approach are needed to fully understand the underlying mechanisms at play in circadian timing efficacy. The study of larger patient populations will enable the validation of our findings across diverse groups. Future research should also address different patient populations and tumors, as the circadian effect may be tumor-specific and more pronounced in certain patients. Ultimately, data from prospective studies will identify which patients can benefit the most from chrono-adjusted immunotherapy and possibly lead to its integration into clinical practice.

## Figures and Tables

**Table 1:** Baseline Patient Characteristics by AM/PM Treatments at Start of Immunotherapy.

	All Patients	Morning Group	Afternoon Group	$\chi^{2}$ Statistic
	(N=73)	(N=48)	(N=25)	(*p*-Value)
**Age** (years)				0.30 (0.581)
Median (range)	70 (29–91)	69 (35–91)	75 (29–86)	
<65	25 (34.2%)	18 (37.5%)	7 (28.0%)	
≥65	48 (65.8%)	30 (62.5%)	18 (72.0%)	
**Sex**				0.31 (0.581)
Female	28 (38.4%)	20 (41.7%)	8 (32.0%)	
Male	45 (61.6%)	28 (58.3%)	17 (68.0%)	
**ECOG PS**				0.00 (≥0.999)
0	48 (65.8%)	32 (66.7%)	16 (64.0%)	
1	25 (34.2%)	16 (33.3%)	9 (36.0%)	
**Melanoma subtype**				1.84 (0.398)
Cutaneous	62 (84.9%)	39 (81.2%)	23 (92.0%)	
Mucosal	2 (2.7%)	2 (4.2%)	0 (0.0%)	
Ocular	9 (12.3%)	7 (14.6%)	2 (8.0%)	

AM (Morning) treatment group: patients with <75% of infusions after 2 p.m. PM (Afternoon) treatment group: patients with ≥75% of infusions after 2 p.m. $\chi^{2}$ test of independence between treatment allocation (AM/PM) and individual characteristics; *p*-values and degrees of freedom (df) reported in parentheses. ECOG PS: Eastern Cooperative Oncology Group performance status.

**Table 2:** Baseline Patient Disease Burden by AM/PM Treatments at Start of Immunotherapy.

	All Patients	Morning Group	Afternoon Group	$\chi^{2}$ Statistic
	(N=73)	(N=48)	(N=25)	(*p*-Value)
**Metastatic Sites** (*N*)				1.71 (0.634)
1	23 (31.5%)	17 (35.4%)	6 (24.0%)	
2	27 (37.0%)	18 (37.5%)	9 (36.0%)	
3	17 (23.3%)	10 (20.8%)	7 (28.0%)	
≥4	6 (8.2%)	3 (6.2%)	3 (12.0%)	
**CNS Metastases**				0.66 (0.417)
Yes	14 (19.2%)	11 (22.9%)	3 (12.0%)	
No	59 (80.8%)	37 (77.1%)	22 (88.0%)	
**LDH** (U/L)				
Median (range)	225 (117–4529)	223 (117–4529)	301 (135–1922)	
≥250 U/L	31 (42.5%)	17 (35.4%)	14 (56.0%)	2.07 (0.150)
≥2 ULN	11 (15.1%)	5 (10.4%)	6 (24.0%)	1.43 (0.232)

AM (Morning) treatment group: patients with <75% of infusions after 2 p.m. PM (Afternoon) treatment group: patients with ≥75% of infusions after 2 p.m. $\chi^{2}$ test of independence between treatment allocation (AM/PM) and disease burden at the start of immunotherapy; *p*-values and degrees of freedom (df) reported in parentheses. CNS metastases: presence of metastases in the central nervous system. LDH: lactate dehydrogenase; U/L: units per liter; ULN: upper limit of normal.

**Table 3:** Toxicities Associated with Immunotherapy by AM/PM Treatments.

	All Patients	Morning Group	Afternoon Group	$\chi^{2}$ Statistic
	(N=73)	(N=48)	(N=25)	(*p*-Value)
**Overall Toxicity**				6.94 (0.225)
No Toxicity	22 (30.1%)	15 (31.2%)	7 (28.0%)	
G1	12 (16.4%)	7 (14.6%)	5 (20.0%)	
G2	27 (37.0%)	16 (33.3%)	11 (44.0%)	
G3	7 (9.6%)	7 (14.6%)	0 (0.0%)	
G4	2 (2.7%)	2 (4.2%)	0 (0.0%)	
N/I	3 (4.1%)	1 (2.1%)	2 (8.0%)	
**Fatigue**				1.66 (0.435)
G1/G2	27 (37.0%)	19 (39.6%)	8 (32.0%)	
G3/G4	0 (0%)	0 (0%)	0 (0%)	
**Cutaneous**				1.98 (0.577)
G1/G2	23 (31.5%)	15 (31.2%)	8 (32.0%)	
G3/G4	1 (1.4%)	1 (2.1%)	0 (0.0%)	
**Endocrine**				3.65 (0.302)
G1/G2	14 (19.2%)	11 (22.9%)	3 (12.0%)	
G3/G4	2 (2.7%)	2 (4.2%)	0 (0.0%)	
**Hepatitis**				3.21 (0.360)
G1/G2	6 (8.2%)	3 (6.2%)	3 (12.0%)	
G3/G4	2 (2.7%)	2 (4.2%)	0 (0.0%)	
**Pneumonitis**				2.96 (0.397)
G1/G2	2 (2.7%)	2 (4.2%)	0 (0.0%)	
G3/G4	1 (1.4%)	1 (2.1%)	0 (0.0%)	
**Renal Insufficiency**				1.55 (0.461)
G1/G2	8 (11.0%)	5 (10.4%)	3 (12.0%)	
G3/G4	0 (0%)	0 (0%)	0 (0%)	
**Uveitis**				2.45 (0.485)
G1/G2	1 (1.4%)	1 (2.1%)	0 (0.0%)	
G3/G4	1 (1.4%)	1 (2.1%)	0 (0.0%)	
**Encephalitis**				2.45 (0.485)
G1/G2	1 (1.4%)	1 (2.1%)	0 (0.0%)	
G3/G4	1 (1.4%)	1 (2.1%)	0 (0.0%)	

Toxicities associated with immunotherapy and respective grades according to CTCAE v5.0 observed in the total study cohort and according to AM/PM treatment groups. AM (Morning) treatment group: patients with <75% of infusions after 2 p.m. PM (Afternoon) treatment group: patients with ≥75% of infusions after 2 p.m. $\chi^{2}$ test of independence between treatment allocation (AM/PM) and toxicity grades; *p*-values and degrees of freedom (df) reported in parentheses. G*x*: grade *x*; N/I: cases for which no information is available; overall toxicity corresponds to the maximum toxicity grade observed.

## Data Availability

The data presented in this study are available on request from the corresponding authors. The data are not publicly available due to ethical restrictions.

## Abbreviations

The following abbreviations are used in this manuscript:

CI	Confidence interval
CNS	Central nervous system
CTC	Circulating tumor cells
CTCAE	Common Terminology Criteria for Adverse Events
CTLA-4	Anti-cytotoxic lymphocyte antigen-4
DC	Dendritic cell
ECOG	Estearn Cooperative Oncology Group
HR	Hazard ratio
ICI	Immune checkpoint inhibitor
Ig	Immunoglobulin
irAE(s)	Immune-related adverse effect(s)
LDH	Lactate dehydrogenase
MHC	major histocompatibility complex
NK	Natural killer
NSCLC	Non-small cell lung cancer
OS	Overall survival
PD-1	Programmed death protein-1
PD-L1	Programmed death ligand-1
PFS	Progression-free survival
PS	Performance status
RECIST	Response Evaluation Criteria in Solid tumors
TAM	tumor associated macrophage
TCR	T cell receptor
ULN	Upper limit of normal
